# Influence of normocalcemic primary hyperparathyroidism in bone density alterations of the jaws in patients with periodontitis

**DOI:** 10.4317/medoral.26958

**Published:** 2024-11-25

**Authors:** Samuel García-Rueda, Cecilia Fabiana Márquez-Arrico, Alberto Herrero-Babiloni, Javier Silvestre-Rangil, Francisco Javier Silvestre

**Affiliations:** 1 Private Dentist, Villena, Spain; 2 University of Valencia, Stomatology Department, Valencia, Spain; 3 University of Minnesota. Research Associate. CIUSSS du Nord-de-l'Île-de-Montréal, Hôpital Sacré-Cœur, Montréal, QC, Canada

## Abstract

**Background:**

Normocalcemic Primary Hyperparathyroidism (NPHPT) is a complex syndrome that causes excess secretion of parathyroid hormone (PTH) from the parathyroid glands. PTH in bone activates the function of osteoclasts, to increase bone resorption and thus increase plasma calcium levels. Given that periodontitis generates osteolytic lesions and has a high prevalence in adults, both pathologies could share etiopathogenic mechanisms, although no studies have been found to date that have investigated this. Therefore, the aim of the study was to evaluate the bone density, periodontal status and biochemistry variables to determinate if there is a relationship between both pathologies.

**Material and Methods:**

A case-control study was carried out with 86 cases (NPHPT) and 87 controls. Bone density was evaluated through computed tomography, measured in Hounsfield units, in seven Regions of Interest. Periodontal status and biochemical variables, such as marker hormones of bone metabolism (25 OH vitamin D and PTH), were analyzed. A Student's t test, bivariate correlations were performed and the OR was calculated.

**Results:**

NPHPT patients are more susceptible to changes in the pattern of bone remodeling due to elevated serum levels of PTH and a decrease in 25OH vitamin D under conditions of normocalcemia. The 58.9% of cases group had periodontitis Stage IV, 27% Stage III, 9.45% Stage II and 8.1% with Stage I. Control group showed a 32% periodontitis Stage IV, 39% Stage III, 8.82% have Stage II and 16.2% Stage I.

**Conclusions:**

There was an association between NPHPT and periodontitis, with patients with NPHPT showing a 1.78 (OR) greater probability of suffering from periodontitis. Our biochemical results showed that the increase in PTH and the decrease in 25OH VIT-D were associated with loss of bone density and these patients presented advanced periodontitis.

** Key words:**Parathyroid hormone, normocalcemic primary hyperparathyroidism, 25 OH vitamin D, periodontitis, bone density, periodontal diseases.

## Introduction

Hyperparathyroidism (HPT) is a complex syndrome characterized by excessive parathyroid hormone (PTH) secretion on the part of the parathyroid glands. It is an infrequent disorder with a low prevalence (1), though advances in the biochemistry, physiology and pathophysiology of both PTH and of calcium and phosphorus, along with studies focused on the determination of calcium in serum, have led to an increasingly earlier diagnosis of HPT. Indeed, the diagnosis is often established in asymptomatic patients that present few signs of disease other than hypercalcemia and elevated PTH levels (2,3).

Parathyroid hormone, also known as parathormone or parathyrin, is a peptide hormone secreted by the thyroid glands, and intervenes in the regulation of calcium and phosphorus metabolism (4).

A rise in PTH produces hypercalcemia (increased blood calcium concentration), while a decrease in PTH results in hypocalcemia (lowered blood calcium concentration) that may lead to muscle spasms (tetany). In addition, PTH regulates the calcium ion concentrations in extracellular fluid, and increases bone resorption by stimulating osteoclast activity, which releases further calcium into the bloodstream (5). The hormone increases the renal proximal tubular reabsorption of calcium released from bone, thereby raising the calcium levels in blood. The effect of PTH is thus the opposite to that of calcitonin (6).

Parathyroid hormone induces the activation or hydroxylation of vitamin D at renal level, transforming it into 1,25 dihydroxycholecalciferol, also known as 1,25 dihydroxyvitamin D (calcitriol). This vitamin in turn activates the transcription of various proteins in the enterocytes, which mediate calcium absorption in the intestine.

Thus, at bone level, PTH activates the osteoclasts to increase bone resorption and thus elevate plasma calcium concentration (7). At kidney level it stimulates calcium reabsorption in exchange for phosphorus, which is eliminated, resulting in hyperphosphaturia and hypocalciuria. In turn, at intestinal level, the hormone acts upon the mucosa, favoring calcium absorption indirectly by elevating the synthesis of 1,25-(OH)2-cholecalciferol (vitamin D3), which in turn acts upon the intestinal epithelium (8).

Of the multiple possible causes of HPT, the most common is the presence of a parathyroid adenoma (9), accounting for 80-85% of all cases of HPT. This is followed in order of frequency by parathyroid hyperplasia (12-16%), which presents two histological variants: chief cell hyperplasia and clear cell hyperplasia. In a lesser percentage of cases primary hyperparathyroidism (PTH1) can be caused by multiple adenomas or double adenomas (2-5%), or even by parathyroid carcinoma (0.5-2% of the cases) (7).

Classically, three clinical forms of hyperparathyroidism have been established: primary hyperparathyroidism (HPT1), secondary hyperparathyroidism (HPT2) and tertiary hyperparathyroidism (HPT3). These forms are differentiated according to the type of underlying cause involved in each case (10).

Normocalcemic primary hyperparathyroidism (NPHPT) is regarded as a new phenotype of the disease, characterized by persistent PTH elevation but with normal blood calcium levels and no identifiable causes of secondary hyperparathyroidism or PTH elevation. (11). The normocalcemic variant of the disorder was first officially recognized on occasion of the third international workshop on the management of asymptomatic HPT, and was defined as the confirmed existence of elevated serum PTH levels in the presence of normal serum calcium concentrations, after rigorous exclusion of all possible causes of secondary hyperparathyroidism (12). It should be underscored that the serum calcium (including ionic calcium) levels must be normal at all times in NPHPT, since hypercalcemia may be intermittent in HPT, and especially in the asymptomatic presentations of the disease.

The underlying biological mechanism of NPHPT is not fully clear. It might constitute a first phase of the disease or represent a particular condition characterized by renal and bone resistance to the action of PTH.

The diagnosis of NPHPT is increasingly frequent, being detected mainly in the course of the evaluation of perimenopausal women with diminished bone mass, or in the evaluation or follow-up of patients with osteoporosis (13).

NPHPT is characterized by a heterogeneous and variable phenotype ranging from the absence of typical features of HPT to symptomatic cases with specific complications (14).

The diagnostic approach first should focus on the exclusion of all possible causes of secondary hyperparathyroidism, particularly vitamin D deficiency (25-OH vitamin D < 30 ng/ml) and renal functional impairment (glomerular filtration rate [GFR] < 60 ml/min based on the CKD-EPI equation). The diagnosis is confirmed on the basis of PTH elevation recorded on at least two occasions, in the absence of hypercalcemia (normal total serum calcium and ionic calcium). However, the determination of ionic calcium poses a series of practical problems that must be taken into account (precision problems, lack of standardization, special sample handling requirements and high cost) (15).

As mentioned, in order to diagnose NPHPT, it is essential to discard the possible causes of secondary hyperparathyroidism. In this regard, vitamin D deficiency is the most frequent cause of high PTH levels and normal serum calcium concentration. The levels defining 25-OH vitamin D sufficiency have not been fully established, though recently deficiency has been defined as < 20 ng/ml (50 mmol/l), with insufficiency corresponding to 25-OH vitamin D levels between 21-29 ng/ml (52.5-72.5 mmol/l), and vitamin D sufficiency as > 30 ng/ml. At the limits of 30-40 ng/ml, the exponential relationship between 25-OH vitamin D and PTH concentrations starts to level off (16). The potential impact of 25-OH vitamin D concentrations in the general population in defining the PTH reference limits is the subject of debate.

Periodontitis is a chronic inflammatory pathology of the periodontal ligament caused by bacteria, which generates a loss of alveolar bone, dental mobility and, in advanced stages, tooth loss (17-21). It is a highly prevalent pathology that mainly affects adults. (17-21). Our research hypothesis focuses the relationship between periodontitis and NPHPT on the alteration of bone metabolism and the osteolytic lesions common in both pathologies. This process of osteolysis in the jaws could be compared to that which occurs in the well-known central giant cell granulomas common in patients with NPHPT, generating osteolytic lesions in the jaw bones (3,9,11,15,16).

Therefore, the aim of this study was to determine the changes in radiographic jaws bone density, the presence of biochemical biomarkers and the severity of periodontal involvement in patients with NPHPT, compared to a control group. As a secondary objective, analyze sociodemographic parameters such as age, gender, oral hygiene habits and tobacco as variables that could influence both pathologies.

## Material and Methods

A case-control study was carried out in age- and gender-matched patients with NPHPT and healthy individuals. Before starting the study, the subjects received written information about the procedures to be carried out. Confidentiality was ensured, and all subjects signed the corresponding consent form. The study was carried out in compliance with the principles of the Declaration of Helsinki (18th General Assembly of the World Medical Association, Helsinki, Finland, 1964), and was approved by the Ethics Committee of Hospital Universitario Doctor Peset (Valencia, Spain)(Reference: CEIm: 31/19).

The medical information and data collected during the study were kept confidential following current regulations on the protection of personal data. The study sample was selected on a random basis in accordance with the eligibility criteria. We thus established a group of patients with NPHPT pertaining to the Departments of Endocrinology and Stomatology (Hospital Universitario Doctor Peset), and a group of healthy volunteers. Both groups were matched for age and gender. The sample size was estimated taking into account the number of annual patients seen in the Department of Endocrinology of the hospital, and consisted of 200 subjects (100 patients with HPT and 100 controls). Assuming a loss rate of 10% and the exclusion of subjects upon applying the screening criteria, the final sample size was 174 subjects (87 patients with NPHPT and 87 controls).

- Inclusion criteria

The included subjects were adults of either gender and between 18-65 years of age. The controls were healthy individuals without any disease history of relevance and who were not using any medication on a chronic basis. All subjects were Caucasian and belonged to the same geographical setting.

- Exclusion criteria

Subjects under 18 or over 65 years of age were excluded, as were patients on hemodialysis and individuals presenting total or partial edentulism precluding the periodontal study (fewer than 14 natural teeth in the mouth). Subjects who had received periodontal or antibiotic treatment in the last 6 months, individuals with active infectious or inflammatory diseases, and patients with degenerative bone disorders, clinically diagnosed diabetes mellitus, or systemic disorders not directly related to NPHPT were likewise excluded from the study.

Healthy individuals seen in the Department of Stomatology of the hospital and with an available computed tomography (CT) study were recruited for inclusion in the control group. A clinical and radiographic periodontal evaluation was made in the Department of Stomatology, with assessment of the patient case history. All these procedures were consistent with routine clinical practice. General data, as well as the medical, drug and dental histories of the patients were documented, and the following explorations were carried out:

- Blood tests.

Bone metabolic markers (25OH vitamin D and PTH) were determined collecting blood samples from a vein in the forearm after a fasting period of 12 hours. Glycosylated hemoglobin (HbA1c), triglycerides, total cholesterol, LDL-cholesterol, HDL-cholesterol, fasting glucose, insulin, and complete blood count were determined as patients routine control, but were not the subject of this study. The blood samples were collected by nurses and analyzed in the laboratory of the Department of Endocrinology.

- Periodontal parameters

Patients were asked about certain habits, with the collection of information on oral hygiene, smoking, eating habits and physical activity. Lastly, a periodontal exploration was carried out using a millimetered North Carolina periodontal probe and a mouth mirror, with recording of the following parameters: probing depth (PD), gingival recession (GR), clinical attachment loss (CAL), gingival index (GI) and plaque index (PI). Tooth identification and notation was made based on the double-digit system adopted by the World Dental Federation (FDI) in 1970.

The latest classification of periodontitis of the 2017 periodontics consensus (22) was used to group the patients into stages (from I-IV) and define periodontitis as early, moderate or advanced.

- Radiological parameters

A CT study was made of the patients with NPHPT, using a low-dose protocol to assess changes in bone mineral density and the presence or absence of variations in maxillary bone quality in these individuals versus the controls. The quantitative tissue density scores in the CT study were expressed as Hounsfield Units (HUs), which are commonly used in three-dimensional (3D) reconstructions and report density on a scale of grays generally ranging from -1000 to +1000. Thus, tissues densities are classified according to their attenuation coefficients as: air (< -1000), water (0), lung tissue (-400 to -600), bone (> +400), soft tissue (+40 to +80) and adipose tissue (-60 to -100). With regard to bone density and quality, Misch established the following classification using HUs (Table 1).

The data obtained were processed using the SPSS version 18 statistical package (license of the University of Valencia), with the calculation of descriptive statistics such as the arithmetic mean, standard deviation (SD) and standard error for quantitative variables, and frequencies and percentages for qualitative variables. The test of homogeneity for two means of independent samples (Student t-test for the comparison of quantitative variables of independent samples) was used, while the chi-square test was employed to assess the homogeneity of two qualitative distributions and compare dichotomic variables. Multiple linear regression models were used to control for confounding variables. The level of significance was established as 5% (α=0.05) in all tests, with statistical significance being accepted for *p* ≤ 0.05.

## Results

- Sociodemographic parameters

The study population consisted of a group of patients with NPHPT and a group of healthy controls. Both groups were matched for age and gender. Taking into account the number of annual patients seen in the Department of Endocrinology, a minimum sample size of 160 individuals was considered (80 patients with NPHPT and 80 controls). Assuming a loss rate of 10% and the exclusion of subjects upon applying the screening criteria, the final sample size was 181 subjects (87 patients with NPHPT and 87 controls).

Females were significantly more predominant in the NPHPT group than among the controls (95.3% versus 79.1%). This is consistent with the widely reported observation that NPHPT is more common among females. The subjects were divided into two balanced groups: *n* = 87 in the NPHPT group and *n* = 94 in the control group. A total of 158 were females (87.2%) and 23 were males (12.8%), with an overall mean age of 68.1 ± 6.0 years (range 60-83).

Based on the full case histories, none of the subjects in either group presented any disease history of relevance, with no diabetes, liver, musculoskeletal or heart diseases, or malignancies, and only four patients in the NPHPT group and three controls presented arterial hypertension (all subjected to pharmacological control). In general, oral hygiene was good, with no significant differences between the NPHPT patients and controls regarding the use of interdental measures of hygiene such as dental floss or interdental brushes. Most subjects brushed their teeth more than two times a day. The frequency of visits to the dentist was very similar in both groups, with the last visit to the dental office having taken place less than a year ago. Likewise, there were no relevant differences between the groups in terms of smoking and alcohol consumption.

- Bone density parameters

Computed tomography was used to measure the bone density of 7 regions of interest (ROIs) corresponding to 7 cephalometric points per patient. The values obtained were used to calculate the mean density per patient, with the stratification of density according to the Misch classification. (Table 1).

**Figure 1:**
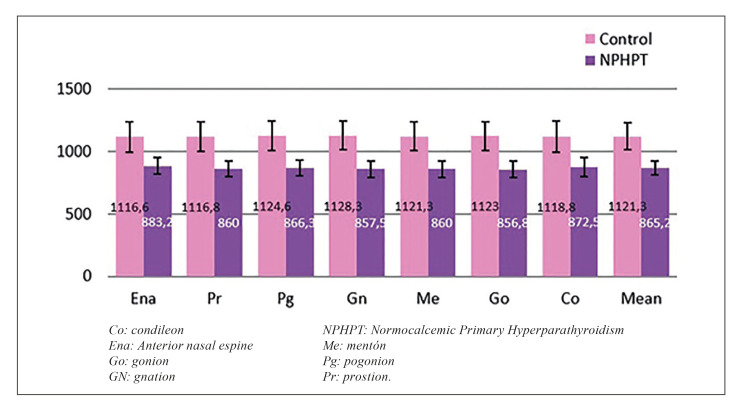
Bone density by region and global average according to group.

Bone density was found to be significantly lower in the NPHPT group than in the control group at all the studied cephalometric points (*p*<0.001). The mean density for all the cephalometric points jointly was likewise significantly lower in the NPHPT group. (Fig. 1, Table 2).

Based on the bone density values obtained, the NPHPT group presented density D2 in 64% of the cases (850-12,450 HUs) and density D3 in 35% of the cases (350-850 HUs). Only 1% of the patients in this group presented bone density D1 (> 1250 HUs) (Table 3).

In comparison to the above, the control group presented bone density D1 in 16% of the cases, D2 in 82%, and D3 in only 2.3% of the cases. Thus, the distribution of bone density according to the Misch classification differed significantly between the two groups (*p*<0.001, Chi2 test). (Table 3).

The measurements were very constant over all the cephalometric points; as a result, the overall mean density was full representative of the bone condition of the jaw. In this respect, the mean bone density in the NPHPT group was found to be 22.8% lower than in the control group.

The bone density in the NPHPT group was significantly lower than in the control group at all the studied cephalometric points (*p*<0.001). The difference was likewise significant on considering bone density for the global points. Thus, the distribution of bone density according to the Misch classification differed significantly between the two groups (*p*<0.001, Chi2 test) (Table 2, Table 3)

The data show that subjects with bone density D1 were only found in the control group, while most of those with bone density D3 were patients with NPHPT. The majority of subjects in both groups presented bone density D2. (Table 3).

- Biochemical parameters

The main hormones that regulate bone regeneration homeostasis and thus osteoclast and osteoblast function in the jaws are PTH, calcitonin and 25OH Vitamin D. The origin of NPHPT is mediated by an excess secretion of PTH, a deficit of 25OH vitamin D, and the presence of normal calcium levels in blood.

The results obtained in relation to the measurement of hormone markers of bone metabolism revealed a significant difference in PTH and 25OH vitamin D levels between the NPHPT group and the controls, with significantly higher PTH levels in the NPHPT group, while 25OH vitamin D was found to be deficient in these patients, as reflected by the following mean values:

It is seen that the PTH levels in the NPHPT group were 51.23% higher than in the control group, while the 25OH vitamin D concentrations were 39.51% lower than in the controls.

- Periodontal parameters

**Figure 2:**
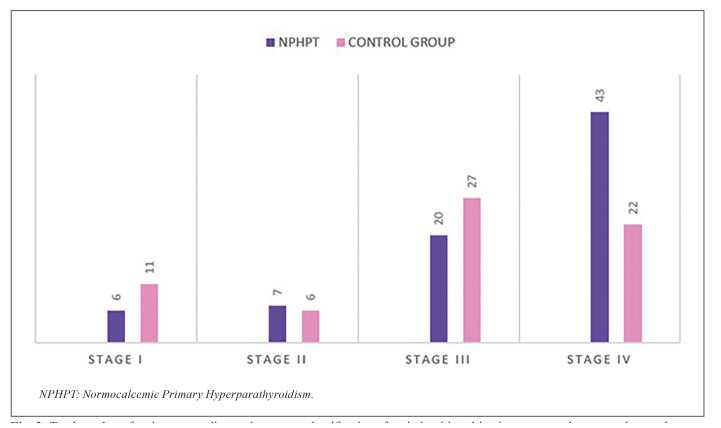
Total number of patients according to the current classification of periodontitis, taking into account the case and control group.

The periodontal study showed that of the 86 patients in the NPHPT group, 76 suffered periodontitis, versus 68 of the controls. There was no periodontal disease in 10 and 16 of the individuals in the NPHPT group and control group, respectively (Fig. 2).

The odds ratio (OR) obtained was 1.78, indicating that the patients with NPHPT were more susceptible to periodontitis than the healthy controls.

The patients with periodontitis were classified by stages (I-IV) according to the degree of clinical attachment loss. The results are reported in the Fig. 2 showing that 58.9% of the patients with NPHPT were in stage IV, 27% in stage III, 9.45% in stage II, and 8.1% in stage I. In comparison, in the control group, the corresponding percentages were 32.4%, 39%, 8.82% and 16.2%, respectively. Thus, stage IV periodontitis was the most frequent presentation in the NPHPT group, versus stage III among the controls, i.e., NPHPT was characterized by the development of more advanced periodontitis (Fig. 2).

## Discussion

Although it has been reported that bone remodeling is modified in patients with NPHPT (23) there have been few studies to date on the changes that occur in the jaws. The association between NPHPT and modifications in bone remodeling of the jaws thus remains unclear, and the underlying mechanisms have not been established (24). On the other hand, no randomized studies have attempted to measure bone density / quality in patients with NPHPT versus healthy individuals, and a review of the literature has revealed no studies on the periodontal disorders found in these patients and their follow-up.

Classically, NPHPT has been associated with loss of the lamina dura of the dental root and with brown tumors of the bone, although recent studies are lacking. Padbury *et al*. (23), in a cross-sectional study, reported that patients with HPT are more likely to have torus mandibularis, and that HPT is characterized by a reduction of the lamina dura of the dental root, a decrease in interdental alveolar bone density, and a decrease in cortical bone density at the gonial angle, associated with expansion of the cortical layer. These authors also postulated that there is a correlation between the plasma PTH levels and the width of the periodontal ligament, and that primary hyperparathyroidism and periodontal disease share the same pathophysiological pathway leading to bone resorption but differ in terms of its underlying etiology and extent (25,26). In our study we analyzed the blood biochemical parameters PTH and 25OH vitamin D and their correlation to the bone density measurements made at the different cephalometric points. Likewise, the values obtained were correlated to the findings of the periodontal study of the patients, where the cause of the decrease in bone density was seen to be 25OH vitamin D deficiency and an increase in PTH levels. The extent of these changes was moreover consistent with advanced stage periodontitis. These results agree with those reported by Sosa Henriquez *et al*., (26).

On the other hand, Palla *et al*., in a recent systematic review on the oral manifestations of primary hyperparathyroidism (27), aimed to identify the oral symptoms found in HPT. They compared the incidence of the disorder stratified according to its three variants: primary, secondary and tertiary. These authors found that high PTH levels frequently induce bone expansion of the jaws, and that there are radiological changes in the lamina dura and in the width of the cortical bone, with lower values in patients with HPT (23). This review also showed that the studies made to date on the oral manifestations of HPT, and the procedures used, lack standardization and the reliable and calibrated diagnostic tests needed to obtain objective results. These observations coincide with the results obtained in our study. There are no cross-sectional or prospective studies on the impact of primary hyperparathyroidism Therefore, the present study could open a new line of research in these field. On the other hand, the lack of studies makes it difficult to compare our results with those found in the literature.

Bone changes traditionally have been reported as oral manifestations of primary hyperparathyroidism, and mainly have been described in patients with advanced HPT, and before the introduction of improved diagnostic techniques (28-30).

Regarding the traditional radiological studies found in the literature (1-3,18,19), it is considered that conventional periapical and panoramic radiographs, as well as lateral cephalometric images, are not very useful in determining bone density, since the lateral cortical layers often mask trabecular bone density, and these two-dimensional techniques are moreover characterized by a degree of image distortion. Although two-dimensional images afford some information, the use of tomographic techniques makes it possible to evaluate bone quality and visualize certain anatomical features of jaw bone such as cortical thickness, the amount of cortical and trabecular bone, and alveolar crest thickness from a three-dimensional (3D) perspective. In addition, these techniques allow millimetric determination of the structural changes of the jaws in patients with primary hyperparathyroidism.

Subjective classifications have been developed for the evaluation of bone tissue based on radiographic data, and in this regard the use of computed tomography (CT) with Hounsfield Units (HUs) is an effective and quantitative technique for the determination of bone density. (31-33). In the present study we used this method to assess the jaw bone density of the subjects in a standardized and precise manner in order to avoid bias (± 1.25 µm).

Based on the bone density measurements obtained, patients with NPHPT were seen to be more susceptible to changes in bone remodeling, due to elevated PTH and reduced 25OH vitamin D levels under conditions of normal blood calcemia. The jaws experienced significant bone changes, with a decrease in the cortical component and particularly in trabecular bone. Specifically, while the controls had a mean bone density of 1124.46 HU, the patients with NPHPT presented a density of 862.18 HU. Thanks to the use of this precise technique we found that patients with NPHPT were more likely to have low jaw bone density than the healthy controls, and moreover had a greater risk of developing advanced stage periodontitis. In effect, 58.9%, 27%, 9.45% and 8.1% of the patients in the NPHPT group presented periodontitis in stages IV, III, II and I, respectively, while in the control group the corresponding Figures were 32.4%, 39%, 8.82% and 16.2%, respectively.

As a limitation of the present study, mention must be made of its cross-sectional or prospective design, which does not allow us to evaluate the impact of NPHPT upon periodontal health over time and establish causal relationships between them.

The results obtained might justify parathyroid gland surgical removal in patients with NPHPT (10-15%), as in the case of individuals with primary hyperparathyroidism, where parathyroid adenomas are the underlying cause of the disorder (85-90%).

Other study have supported our hypothesis, showing HPT to be associated with clear structural modifications (34), including bone alterations at jaw level. Furthermore, as in the case of periodontitis, it has a direct impact upon jaw bone, thus pointing to a two-directional relationship in which patients with HPT and periodontitis are more vulnerable to bone remodeling patterns characterized by lesser bone density.

To date, there is no study available that assesses periodontitis in patients with NPHPT, a fact that makes it difficult to compare our results, although, on the other hand, this study could open a new and interesting field of research.

## Conclusions

Patients with NPHPT were found to have a greater risk of developing periodontitis (OR = 1.78) than the healthy controls, in addition to presenting more advanced stages of periodontitis. In turn, the biochemical findings showed an increase in PTH and a decrease in 25OH vitamin D to be associated with a loss of bone density, and such patients moreover presented advanced stage periodontitis.

## Figures and Tables

**Table 1:** Mish classification. Bone quality according to density measured in Hounsfield Units (HUs).

Density Bone	HU
D1	> 1250 HU
D2	850-1250 HU
D3	350-850 HU
D4	150-350 HU
D5	< 150 HU

**Table 2:** Bone density in Hounsfield Units by region and global average according to group.

Region	Global Average	Group			P value
		Total	Control	NPHPT	
ENA	N	181	94	87	*p*<0,001
	Mean (SD)	999,9 (153,4)	1116,6 (123,5)	883,2 (67,3)	
Pr	N	181	94	87	*p*<0.001
	Mean (SD)	988,4 (159,4)	1116,8 (117,5)	860,0 (63,0)	
Pg	N	181	94	87	*p*<0,001
	Mean (SD)	995,5 (160,7)	1124,6 (119,4)	866,3 (62.9)	
Gn	N	181	94	87	*p*<0,001
	Mean (SD)	992,9 (165,9)	1128,3 (116,3)	857,5 (68,8)	
Me	N	181	94	87	*p*<0,001
	Mean (SD)	990,6 (161,1)	1121,3 (115,5)	860,0 (65,5)	
Go	N	181	94	87	*p*<0.001
	Mean (SD)	989,9 (163,4)	1123,0 (116,3)	856,8 (65,6)	
Co	N	181	94	87	*p*<0.001
	Mean (SD)	995,6 (160,0)	1118,8 (122,4)	872,5 (76,2)	
Total	N	181	94	87	*p*<0,001
	Mean (SD)	993,3 (154,4)	1121,3 (107,9)	865,2(56,6)	

Co: condileon; Ena: Anterior nasal espine; Go: gonion; GN: gnation; NPHPT: Normocalcemic Primary Hyperparathyroidism; Me: mentón; Pg: pogonion; Pr: prostion. SD: Standar desviation. *p-value* results were obteined applying Student T test.

**Table 3:** Bone density according to group expressed in % following Mish classification.

Density Bone	Group						*P value*
	Total		Control		NPHPT		
	N	%	N	%	N	%	
Total	181	100,0%	94	100,0%	87	100,0%	-
D1	14	7,6%	23	25,1%	0	,0%	*p*<0,001
D2	128	73,3%	71	72,6%	55	64,0%	*p*<0,001
D3	39	19,2%	2	2,3%	32	36,0%	*p*<0,001

NPHPT: Normocalcemic Primary Hyperparathyroidism; *p*<0.001, Chi2 test.

## Data Availability

Data will be made available to the editors of the journal for review or query upon request.
